# Progress towards rubella elimination after implementation of rubella immunization for over 20 years in Shandong province, China

**DOI:** 10.1038/s41598-017-18281-2

**Published:** 2017-12-21

**Authors:** Changyin Wang, Zhen Zhu, Qing Xu, Xueqiang Fang, Xiaodong Liu, Ping Xiong, Lizhi Song, Wenbo Xu, Aiqiang Xu

**Affiliations:** 1 Shandong Provincial Key Laboratory for Infectious Disease Control and Prevention, Shandong Center for Disease Control and Prevention, No. 16992, Jingshi Road, Jinan, 250014 People’s Republic of China; 2 WHO WPRO Regional Reference Measles/Rubella Laboratory and Key Laboratory of Medical Virology Ministry of Health, National Institute for Viral Disease Control and Prevention, Chinese Center for Disease Control and Prevention, No.155, Changbai Road, Changping District, Beijing, 102206 People’s Republic of China; 3 Medical school, Anhui University of Science and Technology, Huainan, 232001 People’s Republic of China

## Abstract

The rubella vaccine has been included in the immunization program in Shandong province of China since 1995. Here we observed the rubella vaccine coverage, epidemiology, serosurvey, and virological surveillance data, in order to identify the challenges impeding the progress towards to its elimination following the implementation of rubella immunization over a 21-year period in Shandong province. We first noted that the annual increase in vaccination coverage resulted in decreased rate of rubella incidence, which was maintained at a low level. Second, the average age of rubella patients had shifted to the 15–29-year age group, making this group the main population affected by the rubella virus (RV). Third, more than 90% of the study population were immune to rubella. However, the positive rate of rubella IgG in some cities was relatively lower indicating that an insufficient proportion of individuals had been vaccinated. Finally, the transmission of the genotype 1E RV was gradually interrupted due to the implementation of rubella vaccination. Unfortunately, the endemicity of the imported genotype 2B RV was established due to the pockets with unvaccinated people. Therefore, comprehensive vaccination coverage of the population, combined with high quality monitoring of rubella, is necessary to achieve the rubella elimination goal.

## Introduction

Rubella virus (RV) is the only member of the genus *Rubivirus* within the family *Togaviridae*, and contains a single-stranded positive polarity RNA genome^1^. RV has only one serotype, and consists of two clades (Clade 1 and Clade 2) and 13 genotypes (1a, 1B, 1C, 1D, 1E, 1F, 1G, 1H, 1I, 1J, 2A, 2B, and 2C), according to the systematic nomenclature proposed by World Health Organization (WHO)^2^. Among them, viruses of 4 genotypes, including 1E, 1G, 1J and 2B, are currently the most frequently reported worldwide^3^. In China, virological surveillance for rubella started in 1999, and continuous surveillance data indicate that two RV genotypes, namely 1E and 2B, have co-circulated in recent years^4,5^.

Rubella is usually considered as a mild self-limited illness caused by RV. However, RV has teratogenic potential^6^. If RV infection occurs just before conception and during the first 8–10 weeks of pregnancy, it can be transferred across the placenta causing fetal infection, and lead to serious consequences, including spontaneous abortion, stillbirth, and congenital rubella syndrome (CRS) alongside cardiac defects, cataracts, and hearing impairment, which are of great public health concern worldwide^1^. In China, in order to achieve the WHO goal of rubella control and elimination, national rubella surveillance was formally integrated into the case-based measles surveillance system in 2015, and suspected rubella cases, based on the WHO definition^7^, were reported to the National Notifiable Disease Reporting System (NNDRS). However, national CRS surveillance has not yet been established.

Immunization with live attenuated rubella virus vaccine has been proven to effectively prevent RV infection and further control rubella and CRS. Therefore, the WHO recommended that all countries that have not yet introduced rubella vaccine, and are providing 2 doses of measles vaccine using routine immunization or SIAs, or both, should consider including rubella-containing vaccine (RCV) in their immunization programs^8^. By 2014, RCV had been introduced into 140 countries (72%), and reported rubella cases had declined by 95%, from 670,894 cases in 2000 to 33,068 cases in 2014^8^. RCV was included in the national immunization program in 2008 in China, using both the imported vaccine (RA27/3 strain) and the domestic vaccine (BRD-II strain) nationwide, with the BRD-II vaccine being the most widely used^5^. The BRD-II vaccine was officially approved for use in the 1990s, but only a few provinces and municipalities introduced RCV into their immunization programs at that time^7^.

Shandong province is a developed province with a population of 97.89 million in 2014, according to the National Bureau of Statistics data, which ranks it as the second most populous province in China. Rubella vaccine has been included in the immunization program of Shandong province since 1995^9^. A case-based rubella surveillance system in Shandong province was set up in 1999 and covered all hospitals in Shandong province. All the suspected rubella cases found in hospitals were reported, and the surveillance data flowed from hospitals and county-level Centers for Disease Control and Prevention (CDC) to the Shandong provincial CDC. In this study, we aimed to observe the changes to rubella epidemiology and to identify the challenges in rubella elimination in Shandong province after the implementation of a rubella vaccine immunization strategy over a period of 21 years (1995–2015), and thus, provide a reference for other countries.

## Methods

### Ethical statement

This study was approved at the 2nd session of the Ethics Review Committee of the National Institute for Viral Disease Control and Prevention at the Chinese Center for Disease Control and Prevention, and all methods used in this study were performed in accordance with the relevant guidelines and regulations. Written informed consent from all participants or legal guardians involved in this study was obtained for the use of serum samples for sero-survey or throat swabs for virological surveillance from either the healthy population or from those with clinically confirmed rubella, respectively.

### Rubella immunization program in Shandong province

The rubella vaccine has been included in the immunization program of Shandong province since 1995, and the target age groups have changed over time. In the first stage, from 1995 to 2007, a 2-dose schedule of monovalent rubella vaccine was administered to infants aged 8 months (RCV1) and to children at 7 or 12 years of age (RCV2). In the second stage, between 2008 and 2009, only a 1-dose combined measles and rubella vaccine was administered to infants at 8 months of age (RCV1). In the third stage, from 2010 to the present, a combined measles and rubella vaccine has been administered to 8-month-old infants (RCV1), and the measles-mumps-rubella vaccine was added for children between 18 and 24 months in 2010, in the western region of Shandong, and then extended to the whole province in 2011 (RCV2). From 2008, the rubella vaccine has been free of charge.

### Annual estimated administrative coverage of RCV

Data of the estimated administrative immunization coverage of the first dose of RCV (RCV1), and the second dose of RCV (RCV2), were obtained directly from the routine immunization reporting system of Shandong province from 2002. The numbers of RCV1 and RCV2 doses administered through routine immunization were used as numerators, and the numbers of those in the target population, as reported by the Provincial Bureau of Statistics over the years, were used as denominators. Due to the influence of migrant children (Supplementary Table 1), the estimated immunization coverage of RCV1 and RCV2 may exceed 100%.

### Rubella epidemiological surveillance data

As noted in a previous report^9^, the reported rubella cases and the annual rubella incidence rate from 1999 to 2003 were obtained from the case-based measles and rubella surveillance system. The data between 2004 and 2015 were taken directly from information supplied by the NNDRS, which was established in 2004, when the case-based measles and rubella surveillance system in Shandong Province merged with the NNDRS. The data source was the same for the above two systems, and there was no difference between the systems.

### RV IgG antibody detection

According to the geographical position, population density, climate and social economy, ten cities in Shandong Province were selected for investigation, and furthermore, three villages or communities were randomly selected in each city. All personal information was registered in each village or community, and individuals were selected using a systematic sampling method according to age stratification. Sex was also considered, and males and females were equally likely to be sampled. Based on this, the respondents selected in each selected city were representative. The sample collection of the selected healthy population was carried out through house visits by prefectural epidemiological staff.

Based on the above approach, 2938 serum samples were collected during the 2014–2015 period from the healthy population of 10 cities. This population comprised 8 different age groups, involving 0 to 2 years (359 serum samples), 2 to 4 years (397 serum samples), 5 to 7 years (334 serum samples), 8 to 10 years (325 serum samples), 11 to 14 years (367 serum samples), 15 to 19 years (426 serum samples), 20 to 39 years (549 serum samples), and $\geqq$40 years (181 serum samples).

The SERION ELISA classic rubella virus IgG kit (Serion, Germany) was used to detect the presence of specific rubella IgG antibodies. This kit has already been widely used in national measles and rubella laboratories in China, and has demonstrated good sensitivity and specificity.

It is generally accepted that a measure of $\geqq$4 IU/mL is considered positive for rubella IgG antibodies^10^. Statistical analysis was carried out using SPSS software version 13.0 (SPSS Inc., USA), and statistical methods (Chi-square test) were used to determine the significance of the RV IgG antibody positive rate among these 10 cities and between the different age groups.

### Sample collection, virus isolation and identification, and phylogenetic analysis of RV

In running the rubella routine surveillance system, clinical specimens, including throat swabs and urine samples, were collected from suspected rubella cases for laboratory confirmation in 13 of 17 cities of Shandong province from 2000 to 2015. Cell lines including African green monkey kidney (Vero) cells and Vero cells expressing the signaling lymphocytic activation molecule (Vero/SLAM), were used for virus isolation, respectively, as previously reported^11^. Viral RNA was extracted from the cultured viruses using a commercial QIAamp Viral RNA Extraction Mini Kit (Qiagen, Valencia, CA), based on the kit instructions. Real-time reverse transcription-polymerase chain reaction (real-time RT-PCR) was used to detect the viral RNA with a Takara one-step PrimeScript^TM^ RT-PCR kit (Takara, Dalian, China), as previously reported^12,13^.

After real-time RT-PCR identification, RT-PCR was performed for the positive samples to amplify the WHO standard genotyping window, using a One-Step RT-PCR kit (Qiagen, Hilden, Germany), according to US CDC protocol^12^. After the purification procedure using a QIA Gel Extraction kit (Qiagen, Valencia, CA), the sequences of PCR amplicons were determined bi-directionally with an ABI PRISM^TM^ 3100 Genetic Analyzer (Applied Biosystems, Foster City, CA, USA).Sequence editing was undertaken using a Sequencher version 5.0 (Gene Codes Corporation, Ann Arbor, MI, USA), and a WHO standard genotyping window (739-nt region) of all Shandong RV was obtained. The phylogenetic tree was generated using the neighbor-joining method, with the Kimura 2-parameters model implemented in a MEGA version 5.0^14^.

During the period from 2000 to 2009, 24 sequences of Shandong RV strains were retrieved from the GenBank database. The nucleotide sequences of 38 RV strains isolated during the period 2010 to 2015 were directly submitted to the GenBank database (accession number: KY774388-KY774425). An additional 19 sequences of genotype 1E RVs during the period from 2001 to 2015, and 6 sequences of genotype 2B RVs during the period from 2008 to 2015 from other provinces of China, were downloaded from the GenBank database for phylogenetic analysis.

## Results

### Rubella vaccine coverage estimates

Since 2002, the estimated coverage of RCV1 in Shandong province gradually increased from 64.44% (2002) to approximately 80% during the period from 2006 to 2008. Before 2008, children had to be vaccinated at the expense of the individual, which led to low vaccination coverage. Since 2008, children have been vaccinated free of cost. The coverage was lower (73%) in 2009 due to the switch from the monovalent rubella vaccine to the combined measles and rubella vaccine. Since 2010, a high coverage level has been maintained (>85%), and the percentage of children who received no vaccinations was <3% in 2015. However, the coverage of RCV2 was low (<80%) before 2011, after which it increased and has exceeded 99% since 2012 (Table 1). The same situation occurred in the 10 cities selected for the RV serosurvey study (Supplementary Table 2).

**Table 1:** Administrative estimated RCV immunization coverage from 2002 to 2015 in Shandong province.

Year	Administrative estimated coverage of RCV1			Administrative estimated coverage of RCV2		
	Target number	Number vaccinated	Estimated coverage (%)	Target number	Number vaccinated	Estimated coverage (%)
2002	803,654	517,873	64.44	637,150	312,750	49.09
2003	877,226	555,138	63.28	576,003	278,835	48.41
2004	753,773	552,297	73.27	475,745	202,486	42.56
2005	968,893	732,116	75.56	511,603	294,801	57.62
2006	918,099	756,276	82.37	521,180	355,685	68.25
2007	962,470	805,472	83.69	640,594	414,969	64.78
2008	1,085,680	870,598	80.19	/	/	/
2009	1,155,645	842,094	72.87	/	/	/
2010	1,099,662	1,058,595	96.26	1,030,247	501,372	48.67
2011	1,196,285	1,135,620	94.93	1,129,638	898,350	79.52
2012	1,137,153	1,177,659	103.56	1,062,364	1,223,668	115.18
2013	1,068,279	1,232,021	115.33	1,203,251	1,217,137	101.15
2014	1,349,698	1,184,647	87.77	1,286,261	1,282,047	99.67
2015	1,468,114	1,426,443	97.08	1,158,600	1,280,287	110.50

### The epidemiological data on rubella in Shandong province

**Figure 1:**
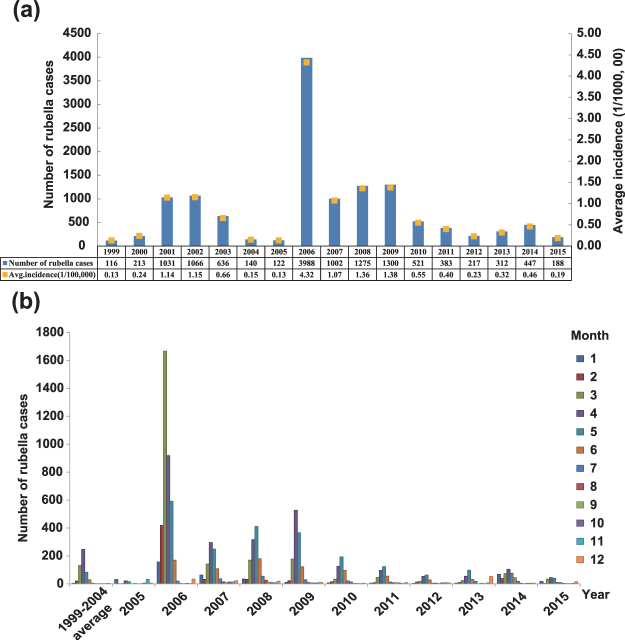
Reported rubella cases in Shandong province by year of onset (**a**) and by month of onset (**b**) from 1999 to 2015.

During the period from 1999 to 2015, 12,957 rubella cases were reported, and more than 90% of those cases were laboratory confirmed. Unfortunately, information on the vaccination history of the cases was not provided with the medical records. The annual rubella incidence was between 0.13/100,000 and 4.32/100,000, of which the highest incidence occurred in 2006. Although the rubella incidence rate has remained at a low level in the past 17 years, the incidence of rubella has peaked on three occasions, between 2001 and 2002 (1.15/100,000), in 2006 (4.32/100,000), and between 2008 and 2009 (1.38/100,000) (Fig. 1a). Following these peaks, the incidence decreased and stabilized at less than 0.5/100,000 over the last 5 years of this study. From 1999 to 2015, cases were reported in all 12 months of the year, and the peak season was usually from March to June (Fig. 1a).

**Figure 2:**
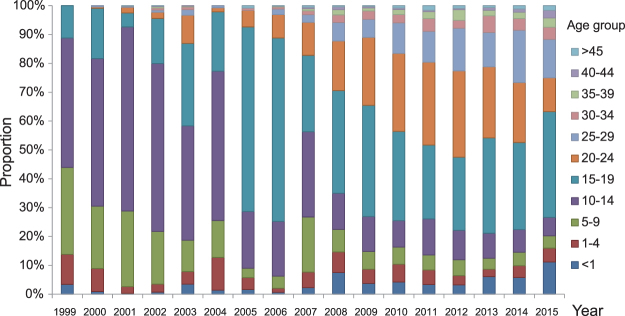
Proportion of rubella incidence in different age groups from 1999 to 2015 in Shandong province.

The proportion of rubella cases aged <15 years deceased from 1999 (89%) to 2009 (27%), and remained at approximately 25% between 2010 and 2015. While the proportion of rubella cases within the 15 to 19-year-old age group increased from 1999 (11%) to 2006 (64%), it has remained steady between 25% and 35% since 2007. The proportion of rubella cases within the 20 to 29-year-old age group also showed a yearly increasing trend from 2000 (1%) to 2012 (45%), and remained at approximately 37% during the period from 2013 to 2014, then decreased to 25% in 2015. Since 2005, the 15 to 29-year-old age group has become the main population with RV infection and the proportion of infected cases in this age group is approximately 70%. The reported proportion of rubella cases aged >30 years old remained low (<10%) between 1999 and 2015 (Fig. 2).

### Rubella serosurvey among the healthy population

**Figure 3:**
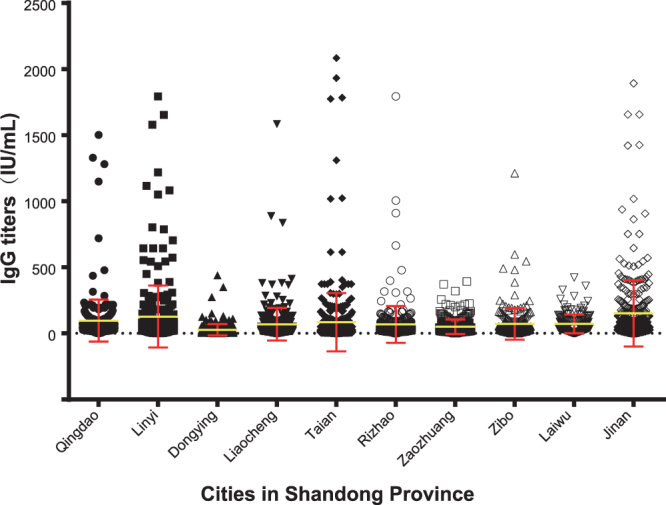
Plot diagram showing the actual IgG titers of rubella positive serum samples.

Among the 2938 serum samples from 10 cities surveyed during the period from 2014 to 2015, 2676 were positive for rubella IgG antibodies, with a total positive rate of 91.08% (Fig. 3). The positivity rate of IgG antibodies in Dongying city (78.12%) was relatively low, while the rates among the other 9 cities were all >89%. There was a significant difference in the positivity rates of IgG antibodies among these 10 cities (Chi-square test, *χ* ^2^ = 157.40, P < 0.01).

The average positivity rate of IgG antibodies in each age group kept relatively high (>85%), and the rate among children aged between 5 and 7 years was the lowest (85.03%). The rate among young adults aged between 15 and 19 years was the highest (96.01%), and the rate was 92.17% among adults aged between 20 and 39 years (Table 2). There was a significant difference in the positivity rates of IgG antibodies among the different age groups (Chi-square test, *χ* ^2^ = 39.05, P < 0.01).

**Table 2:** Rubella IgG detection results in 10 cities of Shandong province from 2014 to 2015.

Age group	Age-standardised proportion positive for rubella IgG										
	Average	Qingdao	Linyi	Dongying	Liaocheng	Tai’an	Rizhao	Zibo	Zaozhuang	Laiwu	Jinan
<2	88.86	95.00	96.00	82.46	84.62	82.26	100.00	100.00	90.91	100.00	86.36
2–4	88.66	97.50	88.00	60.87	91.84	81.63	100.00	97.30	85.71	95.83	94.12
5–7	85.03	100.00	84.00	54.41	92.50	90.91	96.97	100.00	90.48	100.00	91.67
8–10	90.46	92.50	94.00	75.00	94.87	86.96	100.00	94.00	76.67	100.00	92.50
11–14	92.37	97.50	96.00	71.43	85.71	96.43	93.51	100.00	85.71	87.10	97.44
15–19	96.01	100.00	94.00	89.19	100.00	100.00	95.92	98.67	94.23	93.94	94.87
20–39	92.17	95.00	89.14	92.68	94.74	87.50	95.31	97.67	94.59	86.67	89.74
≥40	95.58	95.00	94.07	100.00	100.00	90.63	100.00	85.71	100.00	100.00	94.87
Total	91.08	96.67	91.64	78.12	92.83	89.52	97.38	97.46	89.66	94.46	92.47

Susceptible age groups were also identified in some cities such as children under 14 years old in Dongying city (54.41–82.46%), and 8–10-year age group in Zaozhuang city (76.67%) (Table 2).

### Rubella virological surveillance

**Figure 4:**
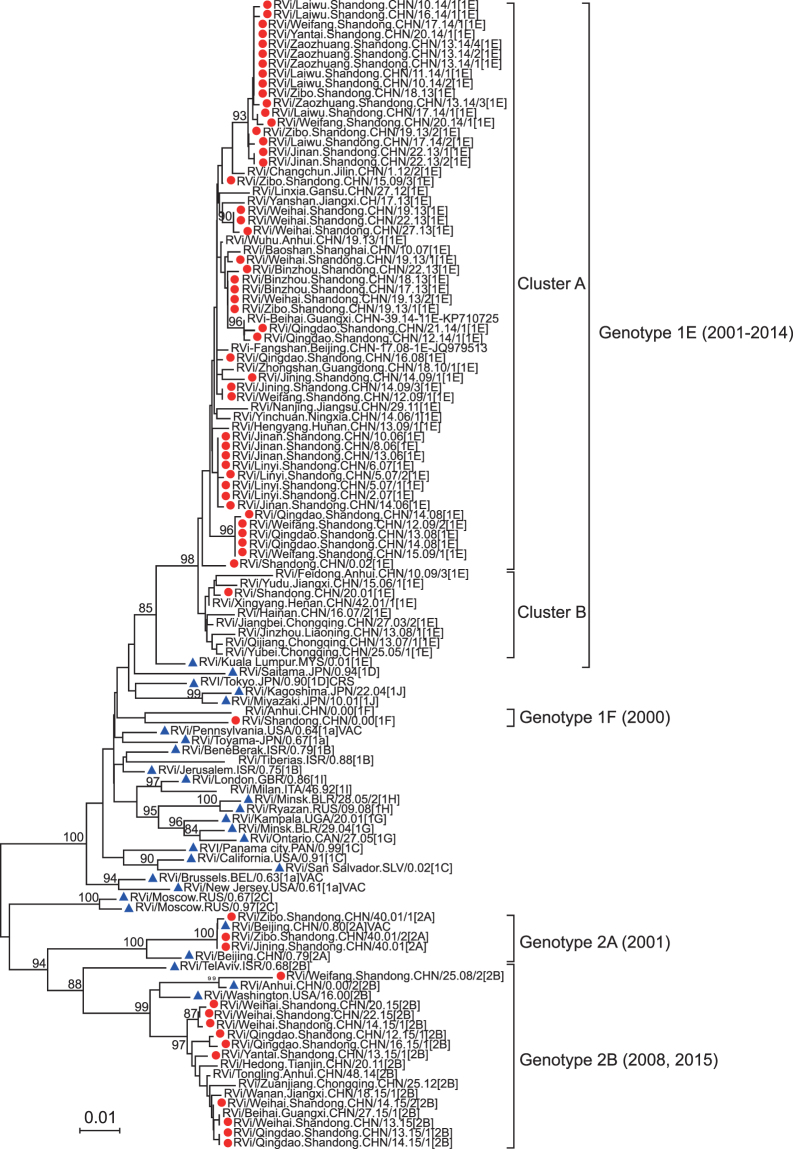
RV genotypes circulating in Shandong province from 2000 to 2015. Neighbor-joining phylogenetic tree of 739nt sequences of rubella viruses isolated in Shandong province from 2000 to 2015. The sequences from Shandong rubella viruses are indicated with a red dot; the sequences of 32 WHO reference strains are indicated with a blue triangle. From 2000 to 2009, 24 sequences of Shandong RV strains were retrieved from the GenBank database (accession numbers AY968210, AY968213, FJ875029-FJ875033, JF702836-JF702840, JF702867, JF702868, JF702870, JQ639404-JQ639412). An additional 19 sequences of genotype 1E RVs were downloaded during the period from 2001 to 2015, and 6 sequences of genotype 2B RVs during the period from 2008 to 2015 from other provinces of China from the GenBank database for phylogenetic analysis (accession number: genotype 1E: JF702819, FJ875044, FJ875066, JF702861, JQ979513, KJ683942, KJ683944, KJ683946, KJ683947, KJ684011, KJ684013, KJ684014, KJ684020, KJ684038, KJ684050, KP710725, KT957752, KT160481, KU884921; genotype 2B: KJ684000, KJ684059, KT160477, KU504549, KU884940, JF702870).

During the years from 2000 to 2015, 72 RV isolates were obtained from either outbreak or sporadic cases in 13 out of 17 cities in Shandong province. For the period from 2000 to 2009, 24 representative RV strains, along with 38 strains for the period from 2010 to 2015, were selected for phylogenetic analysis (Fig. 4). The results showed that the Shandong RV sequences during the years between 2000 and 2015 could be divided into four genotypes, namely 1E, 1F, 2A, and 2B. Apart from one genotype 1E RV isolated in 2001 belonging to 1E-Cluster B, all the other 55 genotype 1E RVs during the period from 2002 to 2014 belonged to 1E-Cluster A. In 2015, 10 strains of genotype 2B RVs from 4 cities were located in a different branch from the RV in 2008 within genotype 2B, and the similarity of nucleotides between them was approximately 95.3% to 96.0%. Three genotype 2A RV strains isolated in 2001 were vaccine-related due to the high similarity (>99.9%) of sequences between these RVs and the Chinese BRD-II vaccine stain. Unfortunately, the vaccination history for these three cases was unknown^9^.

In order to study the correlation of genotype 1E and 2B RVs between Shandong province and other provinces in China, a selection of 19 representative sequences of genotype 1E, from 16 provinces or cities for the years 2001 to 2015, and 5 representative sequences of genotype 2B, from 6 provinces for the years 2008 to 2015, was made for phylogenetic analysis (Fig. 4). The results showed that the genotype 1F virus has disappeared since 2001; both genotype 1E and 2B RVs from Shandong province were intertwined with the RVs from different provinces of China, and there was a good chronological correlation between the strains. Genotype 2B RVs circulating during the years from 2000 to 2008 have disappeared and, since 2011, the imported genotype 2B RV, whose source remains unidentified due to limited epidemiological data has spread in China including Shandong province, resulting in some transmission chains.

## Discussion

Rubella vaccine has been included in the immunization program of Shandong province since 1995. Due to the steady implementation of rubella immunization over the 21 years studied, the annual rubella incidence rate remained low in all age groups, and the natural epidemic cycle of rubella has probably been broken in Shandong province. However, the age distribution of rubella cases has shown significant changes. A similar situation had already been reported in other countries including Greece^15^, Brazil^16^, Costa Rica^16^, and Japan^17^. For example, in Greece, the MMR vaccine was introduced in 1980, and the proportion of susceptible women of childbearing age gradually increased due to the low population coverage (<50%), after which a series of outbreaks occurred. In 1993, 25 infants (24.6/100,000 live births) were diagnosed with defects associated with CRS^15^. Therefore, the countries or regions attempting rubella elimination should maintain a high routine vaccine coverage among children (>85%)^18^, otherwise, there is a possibility of a shift in the age-specific incidence of infection and a greatly increased potential risk of CRS.

In Shandong province, CRS surveillance has not yet been well developed; therefore, the true burden of CRS in Shandong province remains unknown. In order to ensure that progress toward control and elimination could be measured, a CRS surveillance system should be established as early as possible in Shandong province, and throughout China.

Apart from population-based vaccine coverage, serosurvey studies could also be used to ascertain population immunity, monitor susceptibility and determine the age groups for vaccination during supplementary immunization activities (SIAs)^19^. The serosurvey results indicated that, through vaccination for over 20 years, more than 90% of the population (91.08%) were immune to rubella, which is consistent with rubella epidemiological data, and the rubella incidence rate maintained at a low level in recent years. However, the positive rate of rubella IgG in some cities was still relatively lower, such as in Dongying city (78.12%), where there is a large floating population due to it being an economically developed city. Immunization programs need to focus on floating populations to improve the vaccination coverage rate. In addition, although the serosurvey studies indicated that the IgG positive rate for each age group also maintained a relatively high level (>85%), susceptible groups were also identified in some cities, such as the children under 14 years of age in Dongying city. Therefore, in such a region, in addition to maintaining the high routine rubella vaccine coverage among children, appropriate SIAs, or selective vaccination aimed periodically at susceptible individuals, are also important to interrupt RV transmission.

Rubella virological surveillance data could also be used to monitor progress towards the goal of rubella elimination^20^. Shandong rubella virological surveillance was initiated in 2000^9^, with genetic baseline data already established. With the implementation of effective rubella surveillance in recent years, an immune barrier within the population has been gradually established, and the transmission of genotype 1E RV during the years from 2001 to 2014, and of genotype 2B in 2000 and 2008, were likely interrupted. However, a new genotype 2B RV was detected in 4 cities of Shandong province successively from March to June of 2015. This new genotype 2B RV arrived into China in 2011^5^, spreading widely from the east to the west of the country. By 2015, this recently introduced 2B virus had already been found in 23 provinces of China, and it has become the predominant genotype circulating in China, replacing genotype 1E (unpublished data), which indicates that there remain an insufficient proportion of vaccinated individuals. Meanwhile, it is also interesting to study the bio-features of newly imported 2B viruses to see if they gain high transmission ability. Accordingly, continuous virological surveillance is necessary to combat the dynamic epidemic spread of genotype 2B RV in Shandong province, which would also be of benefit in the development of immunization strategies throughout China.

In conclusion, through the implementation of a rubella vaccine immunization strategy over 21 years in Shandong Province, various issues and challenges have emerged. Firstly, although the rubella incidence rate remained low, the acquisition age of rubella infection had already shifted to the 15 to 29-year age group (women of childbearing age), which became the main population infected with RV, and greatly increased the potential risk of CRS. Secondly, an effective CRS surveillance system has not yet been established, which means the true disease burden of CRS remains unknown. Thirdly, inadequate vaccination coverage occurred in some cities of Shandong province, which indicates poorly targeted immunization coverage in these regions. Finally, the transmission of the genotype 1E RV was gradually interrupted due to the implementation of rubella vaccination, but there were still pockets with unvaccinated individuals and, unfortunately, the newly arrived genotype 2B RV had already become endemic.

In order to address the above challenges raised in the process of implementing a vaccination strategy and ultimately reaching the elimination goal, it is necessary to achieve extensive vaccination coverage. This coverage needs to be maintained to establish a stabilized immunization barrier and to interrupt virus transmission, in particular through strengthening the routine infant vaccination schedules and carrying out SIAs among the susceptible population. In addition, a high-quality surveillance system, incorporating clinical and laboratory surveillance, is also necessary to monitor the changes in rubella susceptibility and respond appropriately.

## Electronic supplementary material

Supplementary tables
