# Supplementary material for: Progress towards rubella elimination after implementation of rubella immunization for over 20 years in Shandong province, China
Source: Sci Rep. 2017 Dec 21;7:17982. doi: 10.1038/s41598-017-18281-2 (PMC5740145; doi:10.1038/s41598-017-18281-2)
Supplement: Supplementary file 1 — Supplementary tables [file 41598_2017_18281_MOESM1_ESM.pdf]

Progress towards rubella elimination after implementation of rubella immunization for over 20 years in Shandong province, China

Changyin Wang[1+], Zhen Zhu[2+], Qing Xu[1], Xueqiang Fang[1], Xiaodong Liu[1], Ping Xiong[1], Lizhi Song[1], Wenbo Xu[2,3*], and Aiqiang Xu[1*]

Supplementary Table 1. Information about the migration children in Shandong province during 2009-2015.

City	2009	2010	2011	2012	2013	2014	2015
Jinan	9696	12093	14274	18337	17822	25610	15403
Qingdao	13617	17217	18379	22763	19080	24519	14328
Zibo	4108	4276	4403	6103	5290	6612	4857
Zaozhuang	2824	2538	2954	3826	3961	3724	2736
Dongying	2881	2771	3035	5140	4009	5590	3469
yantai	5700	6606	6693	9562	8987	13575	7114
Weifang	5100	5765	7241	8996	8105	10032	6059
Jining	7336	7649	7897	9482	8881	10025	5891
Tai'an	932	1341	1390	2219	2419	2112	1224
Weihai	3598	3526	3828	5244	4916	7242	4077
Rizhao	2370	2781	2552	2749	2268	2362	1047
Laiwu	837	803	1155	1616	1225	1102	640
Linyi	10486	10670	12136	14818	13822	14688	8923
Dezhou	2555	3037	3033	5319	5625	10902	6262
Liaocheng	8779	6500	6878	8700	6031	6643	3782
Binzhou	2256	2838	2808	3691	3243	4701	3434
Heze	4026	4302	5021	8941	10497	12763	9490
Total	87101	94713	103677	137506	126181	162202	98736

Supplementary Table 2. Administrative estimated RCV immunization coverage of 10 cities, where were selected for RV serosurvey study in this paper.

City	2009		2010		2011		2012		2013		2014		2015	
	RCV1	RCV2	RCV1	RCV2	RCV1	RCV2	RCV1	RCV2	RCV1	RCV2	RCV1	RCV2	RCV1	RCV2
Jinan	75.82	/	96.41	66.33	96.17	92.54	98.78	103.70	108.88	106.02	88.00	97.65	98.68	106.70
Qingdao	64.83	/	95.59	53.14	93.98	83.74	97.98	128.37	100.07	101.47	89.92	107.77	98.00	109.22
Zibo	83.79	/	97.07	64.38	105.50	83.72	105.39	139.37	118.26	115.37	93.63	114.34	96.24	107.17
Zaozhuang	84.09	/	95.62	65.13	101.67	94.12	109.97	112.73	73.80	99.76	96.76	76.01	92.63	76.70
Dongying	77.63	/	81.69	60.72	82.28	80.45	80.91	83.56	81.66	86.84	84.21	83.24	85.74	98.40
Tai'an	84.95	/	101.91	66.09	102.89	96.76	106.52	101.81	96.72	104.03	81.69	90.70	94.63	96.00
Rizhao	68.90	/	98.27	56.01	89.85	93.09	100.25	105.78	106.05	93.33	84.67	96.13	88.73	95.00
Laiwu	86.27	/	99.50	73.01	94.43	84.27	107.16	109.75	104.24	103.03	89.21	94.97	97.65	101.35
Linyi	61.91	/	97.51	57.56	90.16	87.58	106.60	102.65	102.41	108.22	89.62	101.88	96.98	103.08
Liaocheng	67.93	/	76.57	47.03	84.53	81.09	92.37	83.40	102.57	92.90	81.34	83.00	91.33	108.31
